# Effect of Repeated Thermal Shock on Mechanical Properties of ZrB_**2**_-SiC-BN Ceramic Composites

**DOI:** 10.1155/2014/419386

**Published:** 2014-01-16

**Authors:** Gang Li, Hongbo Chen

**Affiliations:** ^1^Applied Science Academy, Harbin University of Science and Technology, Harbin 150001, China; ^2^Beijing Institute of Aerospace Systems Engineering, Beijing 100076, China

## Abstract

ZrB_2_-20 vol.% SiC-10 vol.% h-BN (particles) ceramic composites (ZSB) were fabricated by hot pressing under inert gas protected. ZSB samples with mean size 75 × 55 × 40 mm^3^ were heated using current heating method and then cooled down to low temperature by circulating water. ZSB samples repeatedly went through thermal shock with 10–50 times under various conditions, respectively. Diverse effects on residual strength of ZSB at different experiment conditions (temperatures, thermal shock times, and heating rates) were investigated. The test results indicated that the residual strength of specimen materials all reached the maximum while the temperature was 1600°C and thermal shock number was less than 50 times. Because ZSB samples could not stand the extremely serious hyperoxidation at very high temperature (1800°C), the residual strength of samples decreased sharply. At 1600°C, when the thermal shock times was 20, ZSB samples' residual strength reached the maximum, but it decreased to the lowest point while the thermal shock times was 30. So we argued that the sensitive thermal shock number was 30. Finally, we analyzed the influences on samples residual strength generated by different heating rates at the same temperature and thermal shock number; the results showed that when heating rate was equal to cooling rate, the residual strength of specimen materials reached the maximum.

## 1. Introduction

Zirconium diboride (ZrB_2_) ceramic matrix composites are attractive candidates for hypersonic vehicles' structure members. In application process, they have to support the high temperature difference or the thermal shock due to the high-heat flow produced in reentry and flight, for during the high-speed flight course, the aircraft will have friction with the air and produce the transient state high temperature and high-heat flow [1–4]. So the thermal shock resistance of the ceramic composites has an important role in some applications such as the predication about aerospace materials' span, reliability evaluation, and the safety designs of high-speed aircraft. Materials' thermal shock resistance is the capacity that the material was not failure after being subjected to high-temperature drastic changes. Due to the sensitivity to crack and the intrinsic brittleness of ceramic composites, the material strength would rapidly decrease after the thermal shock and the composites would produce cracks or brittle failure, becoming invalid, and the reliability of material seriously degraded.

Studies have shown that one main factor having influences on material's span was the thermal shock resistance of high-temperature structural ceramic composites [5–8]. So ceramic composite's reliability and thermal shock resistance have been among the main research directions in high-temperature structural ceramic composite fields. Directed towards the high-temperature structural ceramic composites' poor thermal shock resistance, Monteverde and Scatteia [9] tested the thermal shock resistance of MB_2_-SiC_p_ (M = Zr or Hf) composites using single room-temperature water quenching method. The test results were limited to a certain extent because they only considered the influences on material strength under the rapid cooling conditions without under the rapid heating conditions.

In this paper, we investigated the materials' thermal shock resistance under rapid heating condition. The specimens were heated using current-heating method and then were cooled down to low temperature by circulating water. ZrB_2_-SiC-BN composites' residual strength after thermal shock was tested under rapid heating conditions, testing the influences on the thermal shock resistance of sample materials at diverse temperature difference, heating rates, and cycle index, analyzing the reason of material failure and investigating the thermal shock resistance mechanism of ZrB_2_-SiC-BN ceramic composites.

## 2. Experimental

Commercially available ZrB_2_ with a mean size of about 3 *μ*m, SiC with a mean size of about 2 *μ*m, and h-BN with a mean size of about 5 *μ*m were mixed. The powder mixtures of ZrB_2_ plus 20 vol.% SiC and 10 vol.% h-BN (ZSB) were ball mixed for 20 h in a polyethylene bottle using ZrO_2_ balls and ethanol as the grinding media. After mixing, the slurry was dried in a rotating evaporator to minimize segregation. Milled powders were fabricated by hot pressing under inert gas protected in a graphite-die-coated boron nitride at 1900°C for 60 min and 30 MPa of applied pressure. The bulk density of each composition was measured using the Archimedes technique with deionized water as the immersing medium. The relative density was determined by dividing the bulk density by the theoretical density based on a rule of mixture calculation. Flexural strength (**σ**) was tested in three-point bending on 3 mm by 4 mm by 36 mm bars, using a 30 mm span and a crosshead speed of 0.5 mm·min^−1^. Each specimen was ground and polished with diamond slurries down to a 1 *μ*m finish. The edges of all the specimens were chamfered to minimize the effect of stress concentration due to machining flaws.

**Figure 1:**
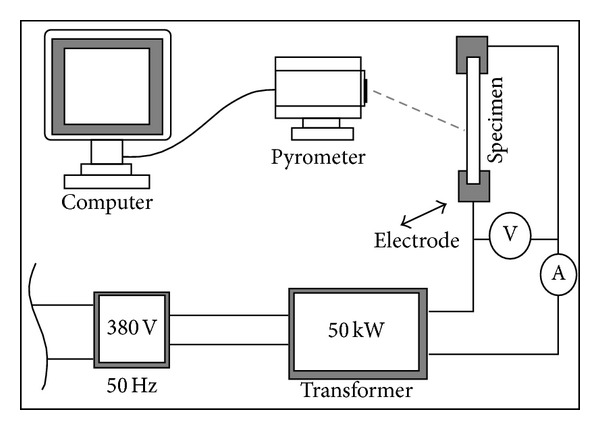
Schematic diagram of current heating test equipment.

The schematic diagram of high current electrical source with power of 50 kW is shown in Figure 1, which was used for the thermal shock test of specimens. The specimen was fixed in Cu electrode and the temperature of the specimen center was measured by multiwavelength pyrometer with measurement range of 1200–2500°C. The voltage for two ends of specimen was maintained at 4 V and the current was increased linearly by program control [10, 11]. The microstructure features of the ZSB composites were observed by scanning electron microscopy (SEM, FEI Sirion, Holland).

## 3. Results and Discussion

### 3.1. Thermal Shock Number Impacts on Thermal Shock Resistance of ZrB_2_-SiC-BN Materials

**Figure 2:**
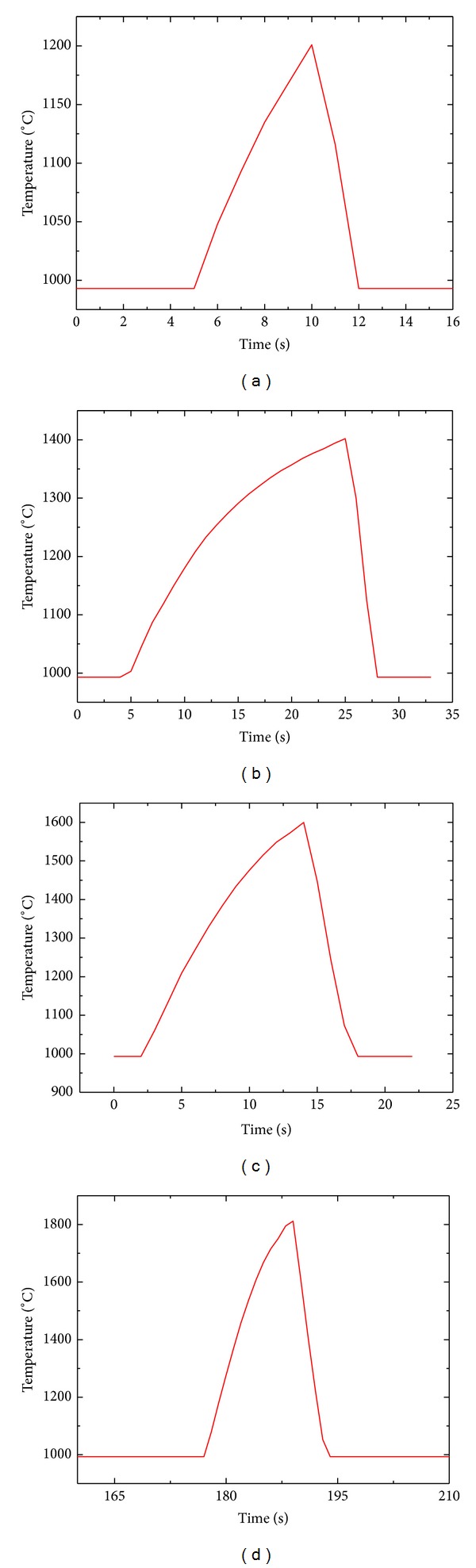
Temperature curve of a single thermal shock: (a) 1200°C; (b) 1400°C; (c) 1600°C; (d) 1800°C.

ZSB sample materials were subjected to thermal shock several times (10 times, 20 times, 30 times, 40 times, and 50 times) by current heating, respectively at four different temperature points (1200°C, 1400°C, 1600°C, and 1800°C), testing the residual strengths of sample materials and observing the changes of sample materials surface morphology and cross-section morphology before and after the thermal shock. The temperature curve of a single thermal shock at four different temperature points (1200°C, 1400°C, 1600°C, and 1800°C) is shown in Figure 2. The variation of sample materials surface temperature can be obtained from Figure 2 quantitatively.

When the thermal shock temperature was 1200°C, the centre temperature of sample materials rose to 1200°C from 1000°C in 5 seconds and fell to 1000°C from 1200°C in 2 seconds with the heating rate being 40°C/s, the cooling rate being 100°C/s, and the current value being 242 ampere. When the thermal shock temperature was 1400°C, the centre temperature of sample materials rose to 1400°C from 1000°C in 20 seconds and fell to 1000°C from 1400°C in 5 seconds with the heating rate being 20°C/s, the cooling rate being 80°C/s and the current value being 238 ampere. When the thermal shock temperature was 1600°C, the centre temperature of sample materials rose to 1600°C from 1000°C in 12 seconds and fell to 1000°C from 1600°C in 4 seconds with the heating rate being 50°C/s, the cooling rate being 150°C/s, and the current value being 254 ampere. When the thermal shock temperature was 1800°C, the centre temperature of sample materials rose to 1800°C from 1000°C in 15 seconds and fell to 1000°C from 1800°C in 6 seconds with the heating rate being 53°C/s, the cooling rate being 133°C/s, and the current value being 278 ampere.

**Figure 3:**
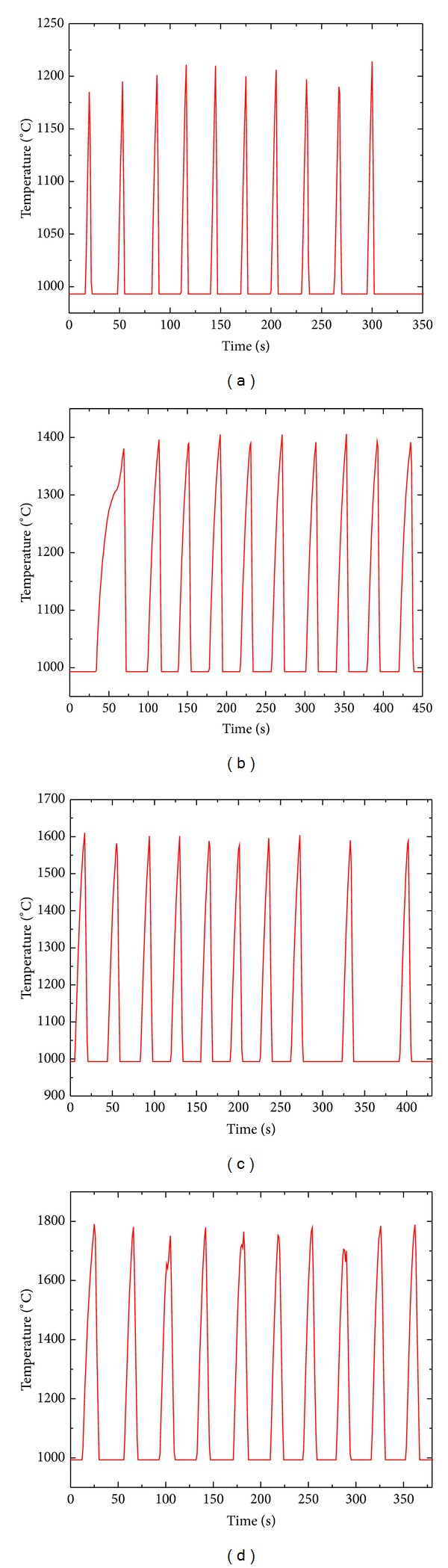
Temperature curve of thermal shock 10 times: (a) 1200°C; (b) 1400°C; (c) 1600°C; (d) 1800°C.

Temperature curves of thermal shock 10 times at four different temperature points (1200°C, 1400°C, 1600°C, and 1800°C) are shown in Figure 3. From Figure 3, we can observe that the maximum of thermal shock temperature basically kept at designed temperature with temperature control was relatively steady and no large fluctuations, the temperature increased linearly, and the heating rate and cooling rate were steady and no large fluctuations. The curves of thermal shock several times (20 times, 30 times, 40 times, and 50 times) corresponded to the curve of thermal shock 10 times, not shown repeatedly.

**Figure 4:**
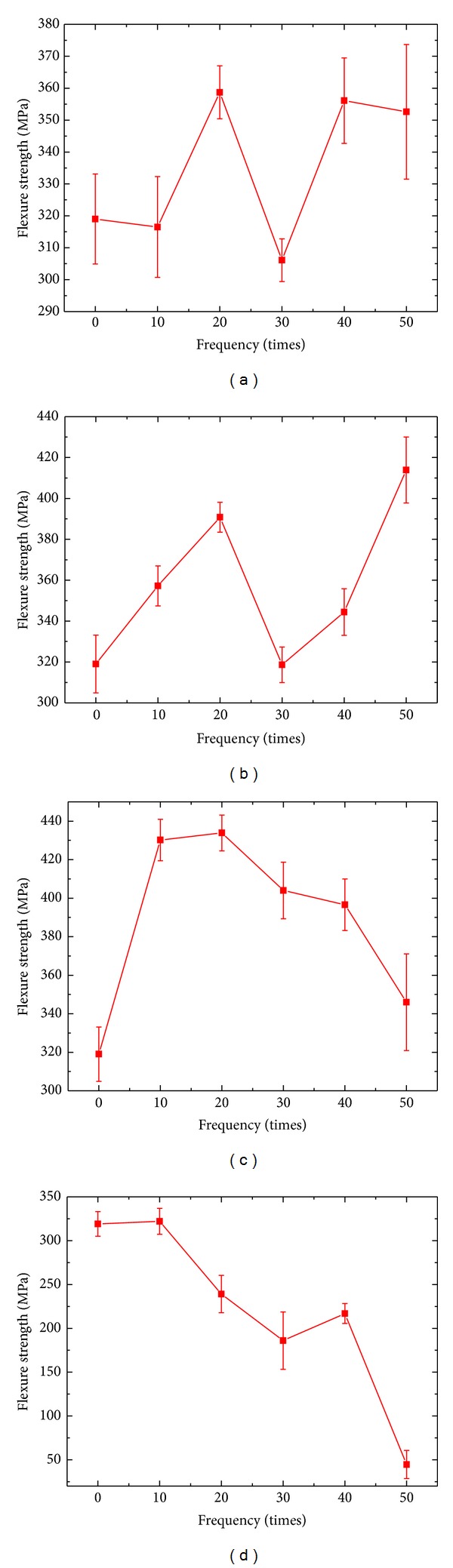
Residual strengths of ZSB materials after thermal shock for different times: (a) 1200°C; (b) 1400°C; (c) 1600°C; (d) 1800°C.

Residual strength curves of ZSB sample materials after thermal shock different times (0 times, 10 times, 20 times, 30 times, 40 times, and 50 times) at four different temperature points (1200°C, 1400°C, 1600°C, and 1800°C) are given in Figure 4. It shows that when thermal shock temperature was 1200°C and thermal shock 10 times, the sample materials' flexural strength which was at a low value decreased a little contrasting to the state of no thermal shock. When thermal shock was 20 times, the sample materials' strength increased with wider amplitude to the maximum value 358.7 MPa. When thermal shock was 30 times, the sample materials' strength fell back with wider amplitude to the level lower than the level of them under thermal shock 10 times. When thermal shock was 40 times, the sample materials' flexural strength rose to the level that was equal to the level of them under thermal shock 20 times. When thermal shock was 50 times, the sample materials' flexural strength had small changes and was weaker than that under thermal shock 40 times.

When thermal shock temperature was 1400°C, the trend of change in the flexural strength of sample materials was jagged rise. It steadily rose before when thermal shock was 20 times. When thermal shock was 30 times, the sample materials' flexural strength significantly decreased to the level of sample materials with no thermal shock. When the thermal shock times were added, the sample materials' flexural strength was continually rising. When thermal shock was 50 times, the sample materials flexural strength was at the maximum value 413.9 MPa. Residual strength of sample materials at thermal shock temperature 1600°C was basically in accord with it at 1800°C and both tended to decrease tardily. In these two conditions, residual strength of sample materials after thermal shock 10 times at 1600°C had a rise of 110 MPa compared to the state with no thermal shock. Then, it decreased with thermal shock times adding and declined to 346 MPa when thermal shock was 50 times but still was higher than the state with no thermal shock 35 MPa. When thermal shock was 10 times, the sample materials flexural strength was equal to that with no thermal shock. Then, the sample materials' flexural strength gradually decreased with the rise of thermal shock times. When thermal shock was 40 times, there was a tiny degree resilient strength 216.8 MPa. When thermal shock was 50 times, the sample materials flexural strength rapidly decreased to 44.6 MPa and the material had lost effectiveness.

Figure 4 shows that even though residual strengths curves at every temperature point had different variation tendencies, there was a common point that residual strengths curves of sample materials had a rise of some extent at other temperature points excluding 1800°C before thermal shock 20 times. Under the condition of few times thermal shock, the surfaces of sample materials were subjected to pre-oxidation treatment, for the sample materials were in the aerobic atmosphere and the heating rate was a little low. From the time shown in Figure 2 (temperature curve of a single thermal shock), we can calculate that the maximum stationary period was two seconds every thermal shock time above 1000°C, so the time of pre-oxidation treatment is 250 seconds when thermal shock 10 times and 500 seconds when thermal shock 20 times. Long pre-oxidation treatment had a positive effect on sample materials flexural strength. So the sample materials flexural strength had a rise when thermal shock less then 20 times. But the sample materials flexural strength had no rise when thermal shock temperature was at 1800°C because ZSB sample materials had much oxidation losses at this temperature. ZSB ceramic composites cannot stand the high temperature by themselves, so the properties of sample materials constantly decreased.

Sample materials residual strengths had a clear decline at four different temperature points when thermal shock 30 times compared to that when thermal shock 20 times, observed from the residual strengths curve. This status was clearer especially at 1200°C and 1400°C. When thermal shock number rose to 40 times, excluding at 1600°C the strength have no rise compared to when thermal shock 30 times, at other three temperature points the materials strengths have rose to some extent. The strength with a clearest resilience rose to the maximum at 1200°C. When thermal shock number rose to 50 times, excluding 1400°C, at other temperature points the materials' flexural strengths all had decreased to some extent, especially at 1800°C the materials' flexural strengths declined to only 44.6 MPa with some sample materials broken and losing effectiveness. Analyzing, thermal shock 30 times reached to the pits of the curve of ceramic composites' residual strengths because the materials' flexural strength at thermal shock 20 times and at thermal shock 40 times both are higher than at thermal shock 30 times and the degree of decline was very clear. So we believed that thermal shock 30 times was ZSB ceramic composites residual strengths' sensitive times during thermal shock.

**Figure 5:**
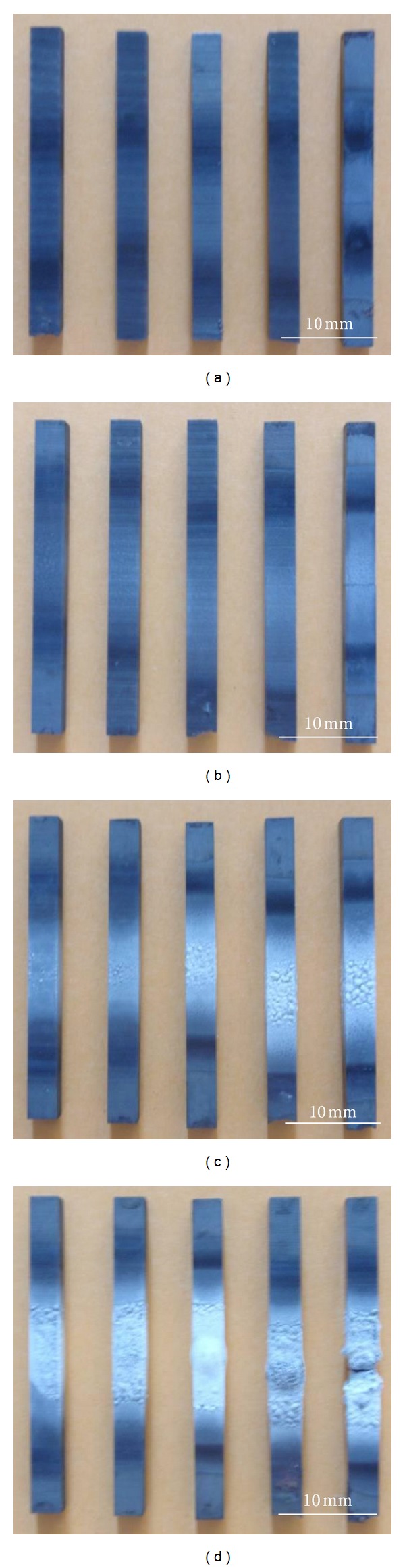
Macro photographs of ZSB materials after thermal shock for 10–50 times: (a) 1200°C; (b) 1400°C; (c) 1600°C; (d) 1800°C.

The surface macrographs of ZSB materials after thermal shock shown in Figure 5 illustrate that surfaces of sample materials have no obvious changes when thermal shock temperature rose to 1200°C. When thermal shock temperature rose to 1400°C, the sample materials' one-third centre was slightly whitish; when temperature rose to 1600°C, the sample materials centre was more whitish with the rise of thermal shock times. When thermal shock number rose to above 30 times, there were obvious oxidation products in the centre of sample materials and the surface had protrusions. When the temperature rose to 1800°C, the sample materials' one-third centre was subjected to serious oxidation treatment producing obvious protrusions and producing fractures at thermal shock 50 times. The test result proved that ZSB ceramic composites cannot tolerate thermal shock above 50 times at 1800°C.

**Figure 6:**
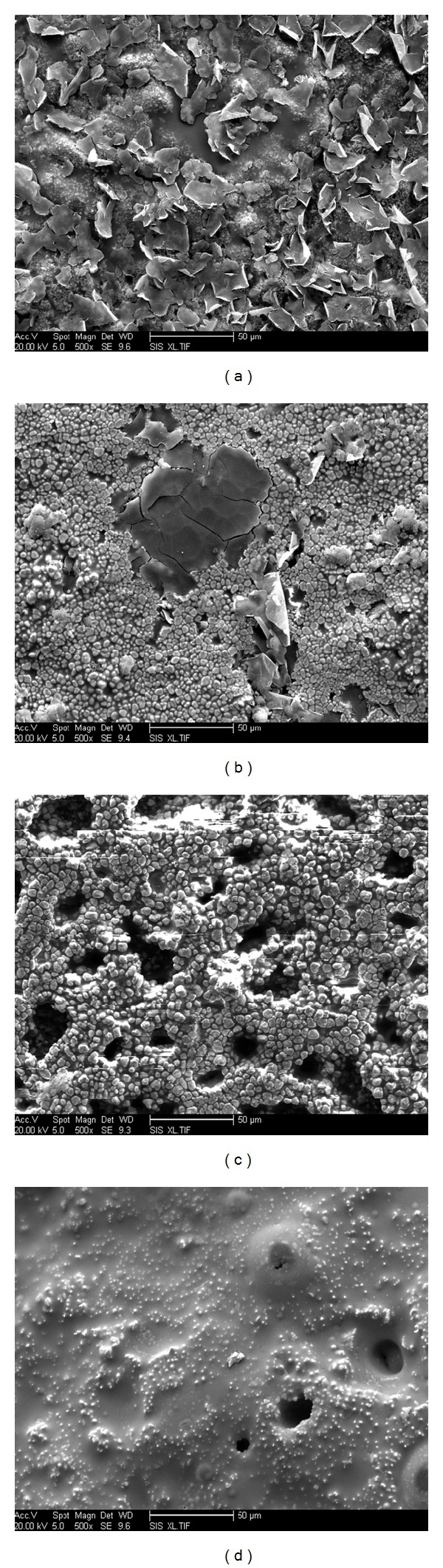
Surface micrographs of ZSB materials after thermal shock for 30 times: (a) 1200°C; (b) 1400°C; (c) 1600°C; (d) 1800°C.

**Figure 7:**
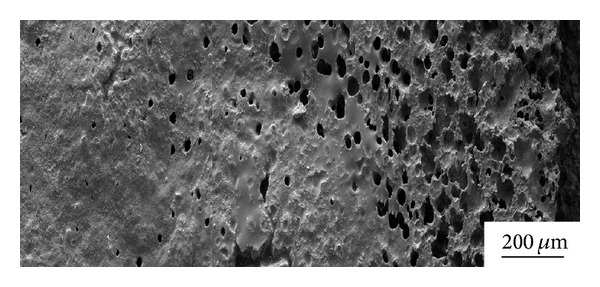
Surface micrographs of ZSB materials after thermal shock for 30 times at 1800°C.

Surface micrographs of ZSB materials after thermal shock 30 times shown in Figure 6 illustrate that expansion scab scale appeared on the materials surfaces when thermal shock temperature was 1200°C and oxidation products with flake appearance adhered to the materials surfaces; when temperature rose to 1400°C, white spotted state oxidation products emerged on the material surface; detected their main component was to be ZrO_2_. And the monolithic material was compact with no obvious pores. When thermal shock temperature went on to 1600°C, pores' number on the sample materials surfaces obviously increased and protrusions emerged on the materials surfaces with relative looseness of material structure. When thermal shock temperature rose to 1800°C, sample materials' one-third centre was subjected to heavy oxidation treatment and had obvious protrusions and the sample materials had a areal deformation when thermal shock 50 times; oxidation products filled small gaps, but large-size pores made materials organization unconsolidated and seriously reduced the material density and mechanical properties. Surface micrographs of ZSB materials' one-third centre after thermal shock 30 times at 1800°C shown in Figure 7 illustrate that getting closer to the centre of sample, pores and notches on sample materials surfaces were more and more, the organization was more unconsolidated, and material oxidation ablation was more serious. When thermal shock number rose to 50 times, fractures emerged on sample materials and sample materials were in failure state.

**Figure 8:**
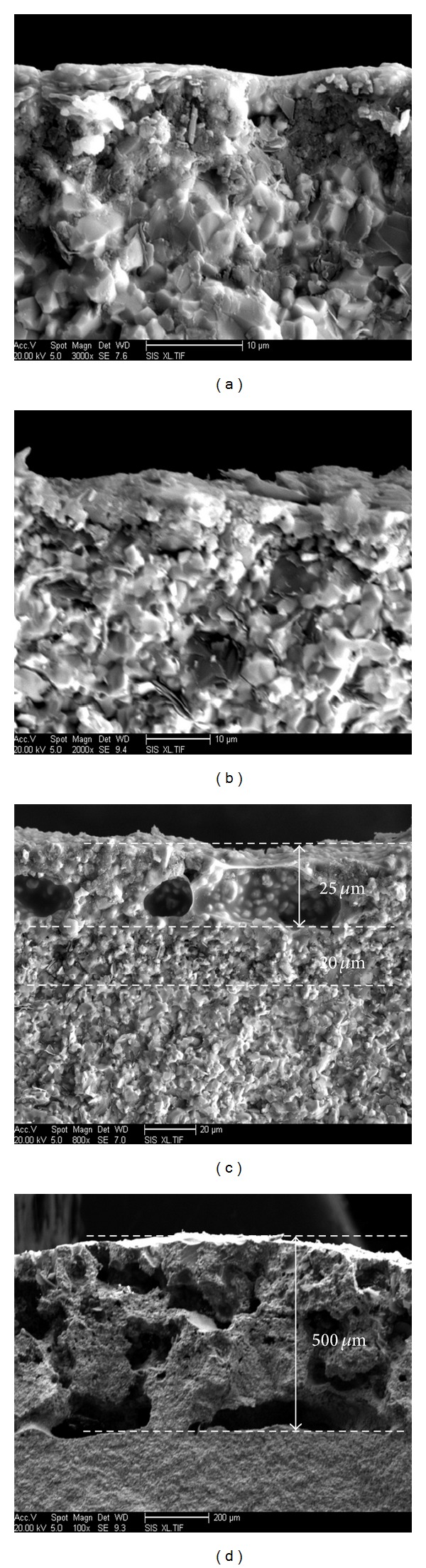
Cross-section micrographs of ZSB materials after thermal shock at different temperature for 30 times: (a) 1200°C; (b) 1400°C; (c) 1600°C; (d) 1800°C.

Cross-section micrographs of ZSB sample materials after thermal shock at four different temperature for 30 times: (a) 1200°C; (b) 1400°C; (c) 1600°C; (d) 1800°C shown in Figure 8 illustrate that only a thin layer of oxide adhered to the matrix surface after material was subjected to thermal shock at 1200°C and 1400°C, matrix organization was integrated, and no pores and notches emerged; when thermal shock temperature rose to 1600°C, material' cross-section oxidation layer produced stratification as shown in Figure 8(c), outer oxidation layer thickness was about 25 *μ*m, interface layer was about 20 *μ*m, and materials' oxidation was obvious at thermal shock temperature 1600°C. When thermal shock temperature was 1800°C, materials surfaces' oxidation was extremely serious, and the oxidation layer thickness arrived to 500 *μ*m as shown in Figure 8(d).

### 3.2. Thermal Shock Temperatures Impact on Thermal Shock Resistance of ZrB_2_-SiC-BN Materials

**Figure 9:**
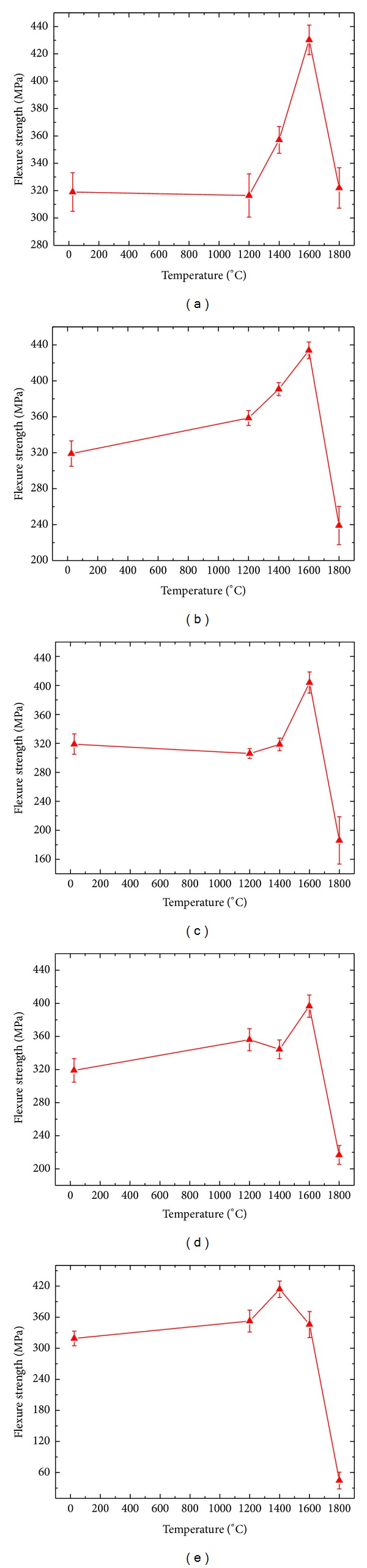
Residual strengths of ZSB materials at different thermal shock temperature: (a) 10 times; (b) 20 times; (c) 30 times; (d) 40 times; (e) 50 times.

Previous section described that different thermal shock times at the same temperature point had effects on ZSB sample materials' residual strength. Present section studied that at the same thermal shock times, different thermal shock temperature made influences on material strength. Sample materials' residual strengths curve at different temperature and same thermal shock times shown in Figure 9 illustrate that sample materials residual strengths' variation tendencies were basically coincident; all firstly increased to the maximum and then rapidly decreased, excluding thermal shock 50 times; at other thermal shock times (10 times, 20 times, 30 times, and 40 times) and 1600°C, the sample materials' strength arrived to the maximum and under 1600°C the strength was gradually rising, but all material flexural strengths rapidly decreased when temperature arrived to 1800°C and the sample materials even fractured with loosing effectiveness. When thermal shock 50 times. It indicated that the material thermal shock limiting temperature was around 1600°C, ZSB ultra-temperature ceramic composites cannot stand 1800°C high-temperature flow thermal shock.

### 3.3. Heating Rates Impact on Thermal Shock Resistance of ZrB_2_-SiC-BN Materials

**Figure 10:**
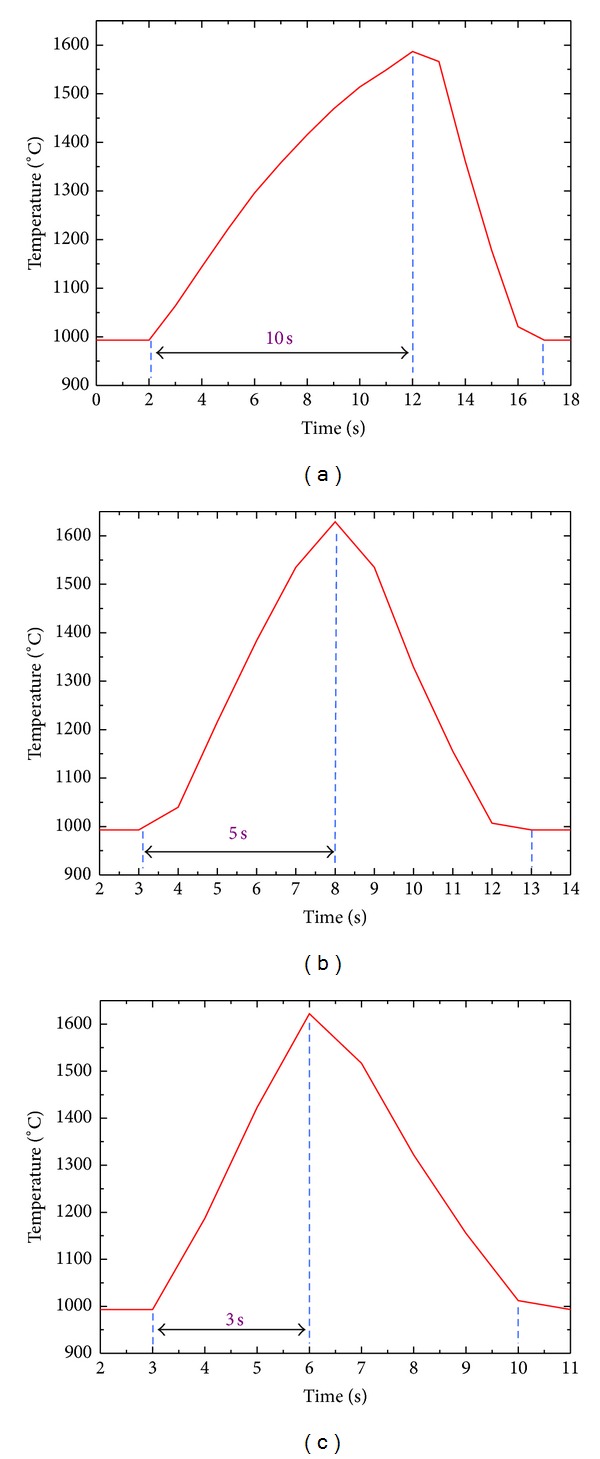
Temperature curves of ZSB materials under different current conditions: (a) 240 A; (b) 280 A; (c) 320 A.

Excluding taking into account that different temperature and thermal shock times made influences on the materials flexural strengths, present section also takes different heating rate created effects on material thermal shock resistance at same temperature and thermal shock number into account.  Figure 10 shows temperature curves of single thermal shock under different current conditions. We can observe current (240 A, 280 A, 320 A) and corresponding heating rate (60°C/s, 120°C/s, 200°C/s). Progresses of drop in temperature all were proceeded using circulated water cooling method after supply suspension and cooling rates all were 120°C/s. Respectively test the material with thermal shock 10 times at above three heating rate, investigating the changes of material residual strengths after thermal shock.

**Figure 11:**
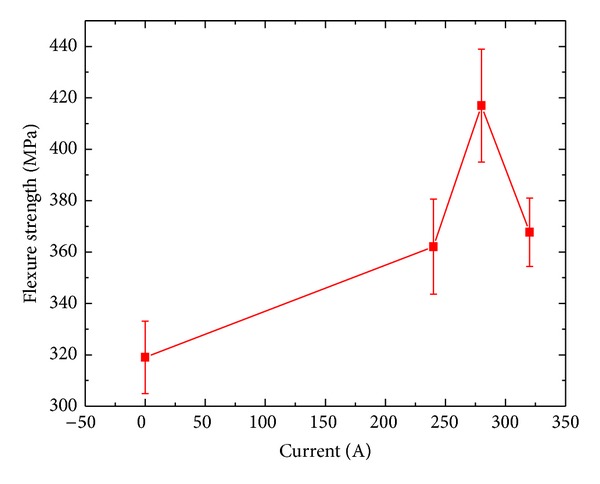
Residual strengths of ZSB materials at different current conditions.

Figure 11 shows ZSB samples' residual strengths at 1600°C after thermal shock 10 times, respectively, at three different heating rates (60°C/s, 120°C/s, and 200°C/s). We can observe that under this condition materials' flexural strengths all were higher than the condition with no thermal shocks and when heating rate was 120°C/s, the material residual strengths arrived to the maximum 417 MPa, while when heating rate is 60°C/s and 200°C/s, the material residual strengths basically were 367 MPa, 12 percent difference. It shows that different heating rate had obvious influences on material residual strengths and when the heating rate was equal to the cooling rate, the materials flexural strength arrived to the maximum value.

For explaining the materials' flexural strength arriving to the maximum value when the heating rate was equal to the cooling rate, we make some brief theoretical analysis. For we cannot perfectly learn about the state of material particles surface and the resilience of particles, the particles with a mean size 2-3 *μ*m can be seen as large micelles, so the powder intergranular acting force was expressed as simple intermolecular forces as follows:

(1) $$\begin{array}{c} f=\frac{A}{r^{\alpha }}+\frac{B}{r^{\beta }}. \end{array}$$

Particles in material did not form the rule structure, so each particle can be seen as they had equal close interaction background, so the force each particle suffering can be expressed as:

(2) $$\begin{array}{c} f=\sum_{i=1}^{N}\left(\frac{A}{{\left(r-r_{i}\right)}^{\alpha }}+\frac{B}{{\left(r-r_{i}\right)}^{\beta }}\right), \end{array}$$

where *N* is particles' ligancy and *r*_*i*_ is the close particles' position vector. So the particles' acting potential field is expressed as:

(3) $$\begin{array}{c} f=\nabla_{r}U. \end{array}$$

When *f* = 0, potential field *U* obtains the extremum; each extremum is correspondent to particles' stationary state and metastable state. According to (2), potential field *U* could have multiple maximum value and minimum value. According to thermodynamics, temperature is a measure of mean kinetic energy of molecule, so when material is during thermal shock, particles would step over the potential hill and then arrive at the metastable state due to the increase of thermal shock intensity. The material intrinsic strength *σ*_*f*_ can be seen as the sum of grain to grain stress in unit cross-section; namely, *σ*_*f*_ = ∑*f*_*t*_.

If the residual strength of material in stationary state is expressed as ${\overset{-}{\sigma }}_{f}$, when particles deviated the stationary state position and entrered the metastable state, the residual strength of local area material would decrease; namely, $\sigma_{f}^{\prime }<{\overset{-}{\sigma }}_{f}$. According to Kingery's thermal shock yield condition, the material will fracture when thermal stress *σ*_*H*_ is greater than material residual strength *σ*_*f*_.

During a complete process of thermal shock, the cooling progress can be seen as recovery progress of heating; namely, during heating progress, particles deviated stationary state and entrered metastable state as a certain probability distribution form, while during the cooling progress, particles return to stationary state again as a certain probability distribution form. So when heating rate is equal to cooling rate, particles' offset distance is more likely equal to recovery distance, material particles are more likely to recover to stationary state, and monolithic material residual strength arrives to the maximum. Nevertheless, the analysis above mentioned is rough; it is still relatively rational to explain the data recorded from the experiment.

## 4. Conclusions

The materials' resistance of thermal shock property under different temperature thermal shock times and heating rate conditions was investigated, respectively. The results indicated that materials' residual strengths all reached the maximum when the temperature was 1600°C and thermal shock times was less than 50. Because samples could not tolerate the extremely serious oxidation at very high temperature as 1800°C, materials' residual strengths obviously decreased. Meanwhile, at the same temperature 1600°C, materials' residual strengths all reached maximum when thermal shock times was 20, but it decreased to a low point when thermal shock times was 30, and no matter the thermal shock times was lower or higher than 30, materials' flexural strengths were all higher than those at 30 times, so we believed that 30 times is the sensitive number of samples thermal shock residual strengths. Finally, testing the different heating rates imposed effects on material residual strengths at the same temperature and thermal shock times, the results showed that material residual strengths reached the maximum when the heating rate was equal to the cooling rate.
